# Outcomes of renal hyperparathyroidism following parathyroidectomy in kidney replacement therapy patients at a tertiary centre in South Africa: A retrospective cohort study

**DOI:** 10.4102/jcmsa.v4i1.316

**Published:** 2026-02-09

**Authors:** Tholakele Sabela, Nontembiso Mhlana, Wilhelmina Conradie, Mogamat-Yazied Chothia

**Affiliations:** 1 Department of Medicine, Faculty of Medicine and Health Sciences, Stellenbosch University, Cape Town, South Africa; 2 Department of Surgical Sciences, Faculty of Medicine and Health Sciences, Stellenbosch University, Cape Town, South Africa

**Keywords:** secondary hyperparathyroidism, chronic kidney disease-mineral bone disorder, parathyroidectomy, hungry bone syndrome, recurrence

## Abstract

**Background:**

Renal hyperparathyroidism is a frequent complication among patients with kidney failure. Data on outcomes following parathyroidectomy in patients undergoing kidney replacement therapy (KRT) remain limited in South Africa (SA). This study aimed to assess postoperative surgical and biochemical complication rates and disease recurrence.

**Methods:**

We conducted a retrospective cohort study of adult KRT patients who underwent parathyroidectomy for renal hyperparathyroidism at Tygerberg Hospital over 7 years. Kaplan-Meier and univariate logistic regression analyses were used to determine predictors of recurrence.

**Results:**

Forty-six patients underwent parathyroidectomy. Two-thirds were female with a mean age of 40.7 ± 8.50 years. Preoperative serum calcium and parathyroid hormone (PTH) concentrations were 2.41 ± 0.27 mmol/L and 176.7 (interquartile range [IQR] 124.4–245.1) pmol/L, respectively. Most patients (80%) underwent subtotal parathyroidectomy. The most common postoperative complication was hungry bone syndrome (HBS), which occurred in 98% of patients, and the postoperative total calcium concentration was 1.63 ± 0.28 mmol/L, observed on day 4 or later. Overall, recurrence of renal hyperparathyroidism occurred in 15% of patients. There were no predictors of recurrence on univariate logistic regression.

**Conclusion:**

A lower recurrence rate was observed in total parathyroidectomy and a lower hypoparathyroidism rate in subtotal parathyroidectomy (SPTX). Hungry bone syndrome was found to be the most frequent postoperative complication, reflecting more advanced disease at the time of surgery.

**Contribution:**

In the future, the focus at Tygerberg Hospital should be on earlier surgical referral considering our limited medical options, and the decision-making regarding the surgical procedure should be discussed in a multidisciplinary team, with a focus on individualised priorities.

## Introduction

Patients receiving kidney replacement therapy (KRT) are at risk of developing chronic kidney disease-mineral bone disorder (CKD-MBD), a spectrum of disorders characterised by abnormalities in bone turnover, mineralisation, volume, growth, and strength because of dysregulated calcium and phosphate metabolism.^1,2^ Metastatic calcification involving vascular and soft tissue structures poses a considerable risk of major cardiovascular events, all-cause mortality, and, in rare cases, calciphylaxis.^3,4,5,6^ A key component of CKD-MBD is renal hyperparathyroidism, encompassing both secondary and tertiary forms.

The diagnosis of secondary and tertiary hyperparathyroidism (tHPT) is based on clinical manifestations and laboratory investigations. Secondary hyperparathyroidism (sHPT) is characterised by appropriately elevated parathyroid hormone (PTH) levels, hyperphosphataemia, and low serum calcium. In contrast, tHPT is characterised by autonomous PTH secretion, leading to elevated PTH levels with normal or elevated serum calcium^5,6^; however, in patients with tHPT who have undergone renal transplantation, serum phosphate levels are often low.^2,6^ Active vitamin D levels are typically reduced in Chronic Kidney Disease (CKD).^2^ Osteopenia or osteoporotic changes can be seen in plain radiographs or bone mineral density scans.

The management of sHPT is primarily preventive, while surgical intervention is reserved for patients who do not respond to medical therapy or who progress to tHPT. The Kidney Disease Improving Global Outcomes (KDIGO) 2017 guidelines recommend that the serum calcium and phosphate be maintained within normal ranges, while PTH is maintained at 2–9 times the upper limit of normal (ULN) for the laboratory assay.^2,7^ First-line treatment of renal hyperparathyroidism includes dietary phosphate restriction, phosphate binders, activated vitamin D analogues, and calcimimetic agents in conjunction with calcium supplementation.^8^ Kidney transplantation is the preferred definitive treatment option for sHPT.^5^ Studies reported that calcimimetic agents have shown good efficacy in controlling the disease and reducing the need for parathyroidectomy; however, because of high costs, these agents are not widely available in the public health sector in South Africa (SA).^9^

Parathyroidectomy is an effective treatment option for controlling complications of renal hyperparathyroidism. An observational study by Milas et al. found that successful parathyroidectomy was associated with improvements in bone density and symptom relief.^10^ In addition, it reduces the incidence of cardiovascular complications and all-cause mortality.^11,12^ Ling Lau et al. reported that parathyroidectomy was inevitably required in about 15% of patients after 10 years and 38% after 20 years of KRT.^13^ Imaging of the neck using ultrasound or technetium-99m (Tc-99m) sestamibi scans is useful for localisation and surgical planning.^5^ The surgical options for renal hyperparathyroidism are subtotal parathyroidectomy (SPTX) and total parathyroidectomy (TPTX). Subtotal parathyroidectomy involves the removal of 3.5 parathyroid glands, leaving a remnant of parathyroid tissue to maintain calcium homeostasis. In contrast, TPTX involves the removal of all four glands and may be accompanied by the reimplantation of parathyroid tissue, also known as auto-transplantation (AT), to prevent permanent hypoparathyroidism.^5,8,14^ There is conflicting evidence on the most suitable surgical option, with a lack of randomised controlled trials.^14,15^ Despite the relative safety of parathyroidectomy, complications of surgery include injury to the recurrent laryngeal nerve and neck haematoma, among others. Acute biochemical complications include hungry bone syndrome (HBS), characterised by severe hypocalcaemia, hypophosphataemia, hypomagnesaemia and hyperkalaemia.^16,17,18^

This study aimed to review post-parathyroidectomy complication rates and disease recurrence in patients with renal hyperparathyroidism in Tygerberg Hospital. In addition, the study compared biochemical outcomes of different surgical approaches to guide recommendations for optimal surgical management of renal hyperparathyroidism.

## Research methods and design

We conducted a retrospective cohort study of adult patients on KRT who were diagnosed with renal hyperparathyroidism and underwent parathyroidectomy at Tygerberg Hospital, a tertiary centre in Cape Town, SA, over 7 years (01 July 2016 to 30 June 2023).

Renal hyperparathyroidism was defined as a PTH concentration > 9 times the ULN for the laboratory assay (> 62.1 pmol/L) despite optimal medical therapy. The data were extracted from the hospital electronic healthcare records and included patient demographics (age and sex), KRT vintage, laboratory parameters, Tc99m-sestamibi imaging, date and type of surgery, and postoperative outcomes. Outcomes included both surgical and biochemical complications, as well as biochemical disease recurrence, defined as a PTH concentration > 9 times the ULN range for the laboratory assay (> 62.1 pmol/L) at 6 months or more post-parathyroidectomy. Hungry bone syndrome was defined as a total calcium concentration < 2.1 mmol/L lasting four or more days postoperatively.^17^

It is important to highlight the protocol followed in our unit during the perioperative period: Preoperative management involves continuing phosphate binders – typically calcium carbonate – and administering high-dose alphacalcidol (3 µg – 4 µg daily) for 3–4 days prior to surgery. The elevated dose is prescribed to mitigate the expected development of HBS. Postoperative management includes monitoring serum calcium six-hourly for the first 48 h and then daily thereafter. The target total serum calcium is ≥ 2.1 mmol/L. Oral calcium carbonate and alphacalcidol are continued, and intravenous calcium is given when the total serum calcium falls below 1.8 mmol/L.

### Statistical analysis

Stata version 16.1 was used to perform data analysis. Descriptive statistics, such as the mean, median, standard deviation (s.d.), and interquartile range (IQR) were used for continuous variables, depending on whether the data had normal or skewed distributions. Counts and percentages were presented for categorical variables. Bar graphs and histograms were used to visualise the variables of interest. Chi-square or Fisher’s exact test was used to compare categorical variables, and the student’s *t*-test was used to compare continuous variables that had a normal distribution. The Mann–Whitney *U* test was used to compare continuous variables that were not normally distributed. Kaplan–Meier survival analysis was also performed to determine recurrence as well as univariate logistic regression to identify predictors of recurrence. Statistical significance was regarded as a *p*-value < 0.05, and the 95% confidence intervals (CI) were used.

### Ethical considerations

Ethical approval was obtained from the Health Research Ethics Committee of Stellenbosch University (approval number: S24/04/093), and a waiver of informed consent was granted.

## Results

### Baseline characteristics

A total of 46 parathyroidectomies for renal hyperparathyroidism were performed during the 7-year study period. Most patients were female (65%), with a mean age of 40.7 ± 8.5 years. The median dialysis vintage was 65 months (IQR 38–96 months) (Table 1).

**TABLE 1:** Baseline demographic characteristics and biochemical data.

Variable	Mean ± s.d.	*n*	%	Median	IQR
**Demographic data**					
Age (years)	40.7 ± 8.50	-	-	-	-
Female (*n* = 46)	-	30	65	-	-
Dialysis vintage (months)	-	-	-	65.00	38–96
**Preoperative biochemistry**					
Total serum calcium (mmol/L) (normal range: 2.15–2.50 mmol/L)	2.41 ± 0.27	-	-	-	-
Serum phosphate (mmol/L) (normal range: 0.78–1.42 mmol/L)	1.89 ± 0.65	-	-	-	-
Serum magnesium (mmol/L) (normal range: 0.66–1.07 mmol/L)	-	-	-	0.85	0.79–0.99
Serum PTH (pmol/L) (normal range: 1.6–6.9 pmol/L)	-	-	-	176.70	124.4–245.1
Serum ALP (IU/L) (normal range: 42–98 IU/L)	-	-	-	190.00	115–288
**Imaging studies**					
Tc99m-sestamibi (*n* = 44)					
Negative	-	5	11	-	-
Indeterminate	-	3	7	-	-
Single hyperfunctioning gland	-	8	19	-	-
Multiple hyperfunctioning glands	-	28	63	-	-
Concomitant thyroid pathology on ultrasound (*n* = 36)	-	6	17	-	-
**Type of operation (*n* = 46)**					
Subtotal parathyroidectomy (≤ 3.5 glands)	-	37	80	-	-
Total parathyroidectomy (4-glands) with AT	-	2	5	-	-
Total parathyroidectomy (4-glands) without AT	-	7	15	-	-

s.d., standard deviation; IQR, interquartile range; PTH, parathyroid hormone; ALP, alkaline phosphatase; Tc99m, technetium-99m; AT, auto-transplantation.

In patients who underwent Tc99m-sestamibi scanning (*n* = 44/46, 96%), 82% demonstrated one or more hyperfunctioning parathyroid glands, whereas 11% showed no evidence of hyperfunctioning glands. Thirty-seven (80%) patients underwent SPTX, while nine (20%) underwent TPTX with (*n* = 2) or without (*n* = 7) AT. The mean preoperative serum PTH, phosphate and total calcium concentrations were 176.7 (IQR 124.4–245.1) pmol/L, 1.89 ± 0.65 mmol/L and 2.41 ± 0.27 mmol/L, respectively (Table 1).

### Postoperative outcomes

**FIGURE 1:**
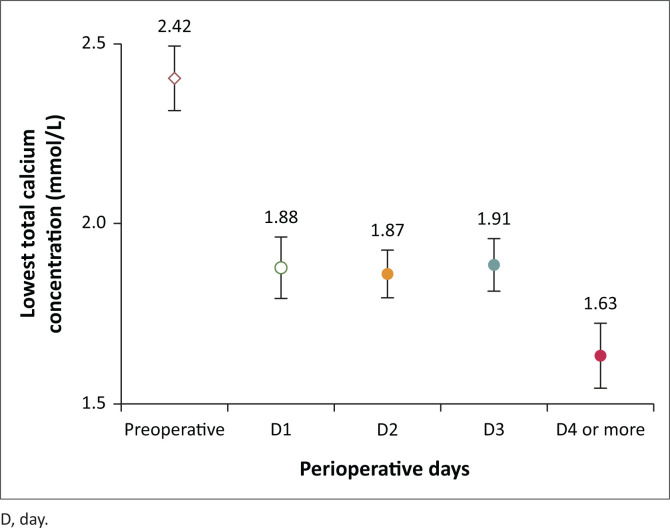
Preoperative and immediate postoperative mean total calcium concentrations from day 1 to day 4 or more.

**FIGURE 2:**
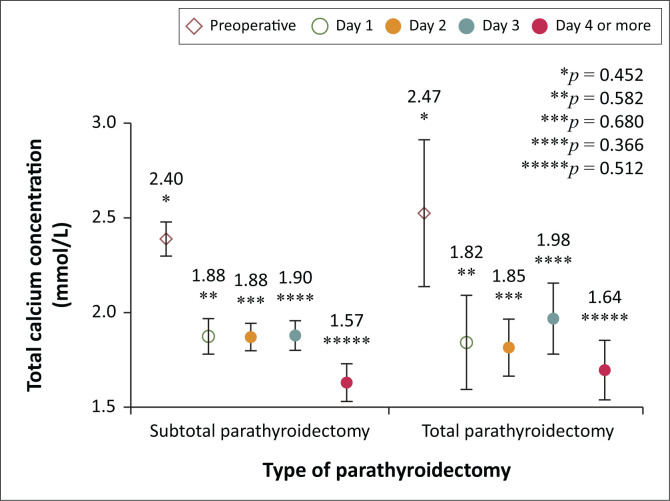
A comparison of the postoperative mean total calcium concentration from day 1 to day 4 by type of parathyroidectomy.

**FIGURE 3:**
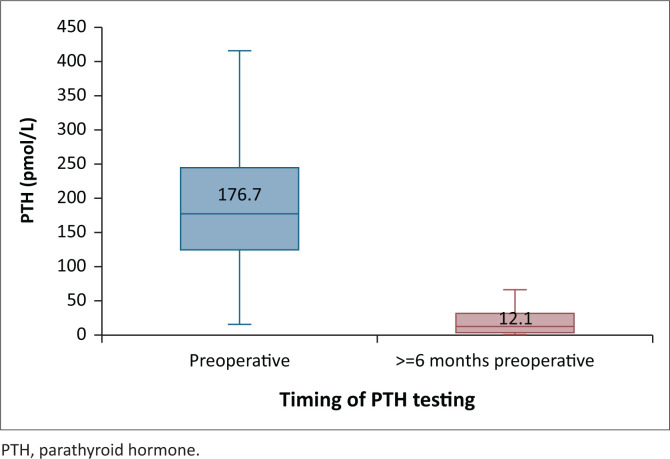
The median preoperative and 6 months or more postoperative parathyroid hormone concentration.

The mean calcium concentration was observed on day 4 or more (Figure 1). There were no statistical differences in the mean total calcium concentration at any time point during the immediate postoperative period between patients who received SPTX or TPTX with or without AT (Figure 2). Serum PTH levels decreased to 12.1 (IQR 2.2–32.0) pmol/L at 6 months or more postoperatively (Figure 3). Parathyroid hormone levels at 6 months or more were < 2 times the ULN for the laboratory assay (< 13.8 pmol/L) in 23/46 (50%), 2–9 times the ULN in 16/46 (35%), and above 9 times the ULN in 7/46 (15%).

Data regarding the histological diagnosis were available for 44 patients. Forty-one patients (93%) were confirmed to have parathyroid hyperplasia, two patients (4%) had a parathyroid adenoma, and one patient (2%) was found to have a parathyroid carcinoma that developed in hyperplastic glands. All patients with indeterminate or negative Tc99m-sestamibi scan results were histologically confirmed to have parathyroid hyperplasia.

The total calcium concentration on day 4 or more was available for 43 patients with HBS, occurring in 42 of 43 patients (98%). The only encountered surgical complication was a neck haematoma requiring reoperation in 3 (7%) patients. Total serum calcium concentration values recorded at 3 months, 6 months, and 1-year post-parathyroidectomy were similar (Table 2).

**TABLE 2:** Postoperative complications, biochemistry data and biochemical recurrence in 44 patients undergoing parathyroidectomy for renal hyperparathyroidism.

Variable	Mean ± s.d.	*n*	%	Median	IQR
**Postoperative complications**					
HBS (*n* = 43)	-	42	98	-	-
Neck haematoma (*n* = 46)	-	3	7	-	-
**Biochemistry data**					
Total serum calcium (mmol/L)					
3-months	2.10 ± 0.48	-	-	-	-
6-months	2.01 ± 0.43	-	-	-	-
1-year	2.09 ± 0.42	-	-	-	-
Serum PTH (pmol/L) at 6-months or more	-	-	-	12.1	2.2–32.0
Serum PTH categories (*n* = 46)					
< 13.8 pmol/L*	-	23	50	-	-
13.8 pmol/L – 62.1 pmol/L**	-	16	35	-	-
> 62.1 pmol/L (recurrence)***	-	7	15	-	-
**Outcome**					
Time to recurrence (months)	-	-	-	7.1	6.5–9.5
Permanent hypoparathyroidism at ≥ 6 months postoperatively, < 13.8 pmol/L (*n* = 46)	-	23	50	-	-

HBS, hungry bone syndrome; s.d., standard deviation; IQR, interquartile range; PTH, parathyroid hormone; ULN, upper limit of normal.

* , PTH level < 2 times the ULN for the laboratory assay;

** , PTH level 2–9 times the ULN;

*** , PTH level > 9 times the ULN. PTH measurements performed at ≥ 6 months postoperatively.

### Recurrence of renal hyperparathyroidism

**FIGURE 4:**
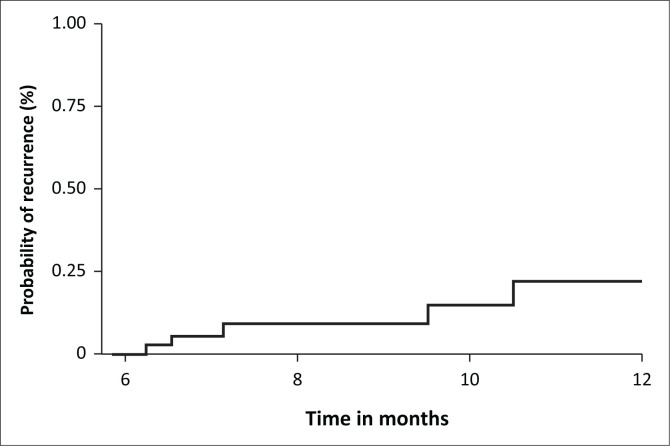
Kaplan–Meier curve for the probability of recurrence at 6–12 months postoperatively.

Biochemical recurrence of renal hyperparathyroidism occurred in 7 (15%) patients at six or more months following parathyroidectomy, with a median follow-up of 7.1 (IQR 6.5–9.5) months (Figure 4) and was observed exclusively in the SPTX group. On univariate logistic regression no predictors of recurrence were identified including age (odds ratio [OR] 0.99; 95% CI 0.90–1.09, *p* = 0.855), male sex (OR 1.50, 95% CI 0.29–7.72, *p* = 0.628), dialysis vintage (OR 0.99, 95% CI 0.97–1.01, *p* = 0.418), preoperative total serum calcium (OR 0.68, 95% CI 0.03–13.75, *p* = 0.798), serum phosphate (OR 1.02, 95% CI 0.29–3.59, *p* = 0.977), serum magnesium (OR 0.96, 95% CI 0.67–1.37, *p* = 0.814), alkaline phosphatase (ALP) (OR 1.00, 95% CI 0.99–1.00, *p* = 0.643), PTH (OR 1.00, 95% CI 0.99–1.01, *p* = 0.968) or vitamin D levels (OR 1.01, 95% CI 0.98–1.05, *p* = 0.464).

Most patients (89%) in the TPTX with or without AT group had a 6-month PTH level below twice the ULN, compared with 41% in the SPTX group (*p* = 0.051) (Table 3). There was no statistical difference in postoperative complications between patients who underwent SPTX and those who underwent TPTX with or without AT (*p* = 1.000).

**TABLE 3:** Comparison of parathyroid hormone and postoperative complications between subtotal versus total parathyroidectomy with or without auto-transplantation.

Variable	Type of operation				*p*-value
	Subtotal parathyroidectomy(*n* = 37)		Total parathyroidectomy with or withoutAT (*n* = 9)		
	*n*	%	*n*	%
**PTH category**	-	-	-	-	0.051
< 13.8 pmol/L*	15	41	8	89	-
13.8 pmol/L – 62.1 pmol/L**	15	41	1	11	-
> 62.1 pmol/L***	7	19	0	0	-
**Postoperative complications**					
HBS	35	95	8	89	1.000
Neck haematoma	1	3	2	2	-

HBS, hungry bone syndrome; PTH, parathyroid hormone; ULN, upper limit of normal; AT, auto-transplantation.

* , PTH level < 2 times the ULN for the laboratory assay;

** , PTH level 2–9 times the ULN;

*** , PTH level > 9 times the ULN.

## Discussion

Despite the global trend of decreasing parathyroidectomy rates because of the advent of calcimimetics, surgical intervention in renal hyperparathyroidism remains crucial for managing cases at our centre because of the lack of access to this class of drugs.^15^ Over the 7-year period, only a small number of patients underwent parathyroidectomy for renal hyperparathyroidism at our centre. This raises concern about potential underdiagnosis or delayed referral. However, the low number of procedures may also reflect the limited number of new patients starting KRT, as dialysis capacity at our centre has remained unchanged over the past two decades.

Most of our patients had SPTX, and only two had TPTX with AT. The type of operation performed for renal hyperparathyroidism depends on various factors. Patient-related factors such as ongoing dialysis, a younger age, and poor dietary and medication adherence, disease-related factors such as a higher PTH, nodular gland morphology and significant bone involvement, and system-related factors including less reliable follow-up and the availability of kidney transplantation might necessitate more aggressive surgery. With SPTX and TPTX with AT, the risk of recurrence is higher, whereas TPTX alone carries a greater risk of permanent hypoparathyroidism.^8,14,15^

A study from the American College of Surgeons – National Surgical Quality Improvement Programme (2005–2013) that included 1 130 patients reported that nearly 70% have undergone SPTX.^14^ In contrast, a Japanese registry study (2010–2013) involving 826 patients who underwent parathyroidectomy found that 75% were treated with TPTX with AT.^19^ The difference in decision-making may be attributed to longer survival and lower kidney transplant rates among Japanese dialysis patients compared to their United States counterparts.^14^ Support for the Japanese approach comes from a 2017 systematic review and meta-analysis of 11 studies involving 1108 patients, which found that TPTX, with or without AT, had comparable outcomes for surgical complications, all-cause mortality, persistence of sHPT, and symptomatic improvement.^20^ Total parathyroidectomy was associated with a lower risk of recurrence and a reduced need for reoperation compared to TPTX with AT but had a higher rate of permanent hypoparathyroidism.^20^

The recurrence rate of renal hyperparathyroidism after parathyroidectomy is variable in the literature, ranging from 5% to 20%.^21,22,23^ The recurrence rate is influenced by follow-up duration, ongoing renal stimulus, the definition of recurrence, and surgical technique. Our findings demonstrated improvements in biochemical parameters, with a recurrence rate of 15% at a median of only 7.1 months postoperatively. Recurrence occurred mostly in patients undergoing SPTX, although one case of recurrence after previous TPTX with AT was excluded from the analysis. This finding is consistent with other retrospective studies^20,24^ Subtotal parathyroidectomy has a 3.3-fold greater risk of recurrence than TPTX with or without AT, and TPTX without AT has a lower recurrence rate than with AT.^22,25^

We did not identify any predictors of recurrence; however, these may include a dialysis vintage of more than 3 years, postoperative PTH > 11.3 pmol/L, and a postoperative phosphorus concentration of > 1.91 mmol/L.^26^ Recurrence is also increased in the presence of supernumerary glands and late referrals with higher PTH.^21,27^ Our patient cohort had many of these factors, including SPTX, late referral with dialysis vintage of > 5 years before parathyroidectomy, and high median PTH and median ALP. These findings highlight the crucial need for early referral, careful patient selection and tailored surgical approaches to minimise recurrence.

Subtotal parathyroidectomy is recommended when kidney transplantation is imminent because of its low risk of recurrence following transplant. A small study comparing outcomes in patients treated with SPTX or TPTX with AT before kidney transplantation found no recurrence in those who underwent SPTX, whereas recurrence and hypoparathyroidism rates were 4% and 15%, respectively, in those who received TPTX with AT.^28^ At our centre, the shortage of deceased donors and limited access to surgical theatres contribute to low transplant rates, which may be another factor contributing to the high recurrence rate in our cohort.

The most frequent postoperative complication in our study cohort was HBS despite our perioperative management protocol, affecting nearly all patients. Goldfarb et al. reported that 28% of 79 patients who had a parathyroidectomy for sHPT developed HBS, which was defined as additional days of hospitalisation or readmission requiring intravenous calcium supplementation because of symptomatic hypocalcaemia.^16^ Risk factors for HBS included age < 45 years, lower preoperative total serum calcium concentration (< 1.75 mmol/L), late referral with higher PTH, higher body weight, higher ALP, and male sex.^17,21,29^ Our patients were relatively young, with many presenting with tHPT evidenced by normocalcaemia or hypercalcaemia with hyperparathyroidism, suggesting delayed surgical referral. Limited data exist on the development of HBS in patients with tHPT, as PTX is typically performed once sHPT becomes refractory to medical therapy in high-income countries. A study in kidney transplant recipients reported that 40% of patients who underwent parathyroidectomy for tHPT developed postoperative hypocalcaemia, defined as requiring calcium supplementation 6 months post-surgery.^28^ This suggests that tHPT may be an additional risk factor for HBS. The high rate of HBS reported in this study is likely a function of our higher serum calcium threshold. Using symptomatic hypercalcaemia along with the need for intravenous calcium supplementation as diagnostic criteria may have yielded lower rates, consistent with the findings of Goldfarb et al.^16^

In our study, a greater proportion of patients who underwent TPTX had PTH levels less than twice the ULN during postoperative follow-up compared with those who underwent SPTX. Permanent hypoparathyroidism is more common in TPTX without AT than with TPTX with AT and STPX.^1,22,23,25,30^ Immediate symptoms of hypoparathyroidism relate to hypocalcaemia with paraesthesia, cramps, tetany and seizures, significantly influencing quality of life. Other longer-term effects include adynamic bone disease with increased fracture risk, and cardiovascular effects with vascular and valvular calcification, further raising cardiovascular morbidity and mortality.^31,32,33^

Limitations of the study include its retrospective design, single-centre setting, and small sample size. This may have resulted in low statistical power when comparing SPTX to TPTX with or without AT. Potential confounders such as dialysis modality, type of medical therapy, and adherence to postoperative treatment were not captured in our data collection. Since only two patients who underwent TPTX had AT, we were unable to stratify by AT status, which may have introduced additional confounding. Furthermore, as recurrence was defined using biochemical criteria, the rate might have differed if symptomatic recurrence or the need for reoperation had also been included. As with most retrospective studies, missing data may have introduced bias; however, few variables had missing data points. We could not perform multivariate logistic regression analysis for predictors of recurrence because too few patients reached this outcome, which may have risked overfitting the model. As this was a single-centre study, the results may not be generalisable because of differences in patient demographics and/or treatment protocols; however, it offers initial insights and provides a basis for a more comprehensive or larger study in the future.

## Conclusion

A lower recurrence rate was observed in TPTX and a lower hypoparathyroidism rate in SPTX. HBS was found to be the most frequent postoperative complication, reflecting more advanced disease at the time of surgery. In the future, the focus at our centre should be on earlier surgical referral considering our limited medical options, and the decision-making regarding the surgical procedure should be discussed in a multidisciplinary team, with a focus on individualised priorities.
